# Antimicrobial activity of Ti-ZrN/Ag coatings for use in biomaterial applications

**DOI:** 10.1038/s41598-018-20013-z

**Published:** 2018-01-24

**Authors:** Anthony J. Slate, David J. Wickens, Mohamed El Mohtadi, Nina Dempsey-Hibbert, Glen West, Craig E. Banks, Kathryn A. Whitehead

**Affiliations:** 1 Microbiology at Interfaces Group, School of Healthcare Sciences, Manchester Metropolitan University, Chester Street, Manchester, M1 5GD UK; 2 School of Science and the Environment, Manchester Metropolitan University, Chester Street, Manchester, M1 5GD UK; 3 ESR Technology, Birchwood Park, Warrington, UK; 4 Surface Engineering Group, School of Engineering, Manchester Metropolitan University, Manchester, M1 5GD UK

## Abstract

Severely broken bones often require external bone fixation pins to provide support but they can become infected. In order to reduce such infections, novel solutions are required. Titanium zirconium nitride (Ti-ZrN) and Ti-ZrN silver (Ti-ZrN/Ag) coatings were deposited onto stainless steel. Surface microtopography demonstrated that on the silver containing surfaces, *S*_*a*_ and *S*_*v*_ values demonstrated similar trends whilst the *R*_*a*_, average height and RMS value and *S*_*p*_ values increased with increasing silver concentration. On the Ti-ZrN/Ag coatings, surface hydrophobicity followed the same trend as the *S*_*a*_ and *S*_*v*_ values. An increase in dead *Staphylococcus aureus* and *Staphylococcus epidermidi*s cells was observed on the coatings with a higher silver concentration. Using CTC staining, a significant increase in *S. aureus* respiration on the silver containing surfaces was observed in comparison to the stainless steel control whilst against *S. epidermidis*, no significant difference in viable cells was observed across the surfaces. Cytotoxicity testing revealed that the TiZrN coatings, both with and without varying silver concentrations, did not possess a detrimental effect to a human monocyte cell line U937. This work demonstrated that such coatings have the potential to reduce the viability of bacteria that result in pin tract infections.

## Introduction

The definition of a biomaterial is any type of material that is used to produce a pharmacologically inert device, which can then aid or replace a function of the body, in a safe, reliable and physiological-acceptable manner^1^. The use of biomedical implants has risen at an exponential rate, due to a variety of factors such as aging population and due to a range of congenital diseases and injuries (including fractures, breaks and excessive strains)^2^. This, coupled with the substantial concern surrounding antimicrobial resistance, due to the overuse of antibiotics means that the development of alternative methods to prevent and reduce infection rates are of paramount importance^3,4^. In 2011, 4% of patients in U.S. acute care hospitals developed at least one healthcare associated infection, with one of every four infections being directly related to a device^5^.

Pin-tract infections are the most commonly expected problem when utilising external bone fixation pins. Left untreated, pin-tract infections will progress, leading to mechanical pin loosening and ultimately the loss of structural integrity/stability of the fixator pin-bone construct^6^. The development of pin-site infections are influenced by a number of patient-specific risk factors, including the surgical technique used, the use of prophylactic antibiotics and the post-operative pin care protocol^7^. This includes the overall maintenance of the external fixation devices (e.g. cleansing, dressing changes and showering)^7^. If left to progress, a pin tract infection can result in discomfort to the patient, additional cost due to treatment regimes (care, cleaning and replacement of device, increased use of drug delivery and antibiotics), as well as the development of other medical conditions including osteomyelitis, septic arthritis, toxic shock syndrome and bacteraemia, with the latter sometimes resulting in death^8^. The failure of medical implants to resist colonisation by pathogens and the rate of infection, is dependent upon a variety of factors. These factors include the presence of pathogenic/opportunistic bacteria surrounding the implant, the surface characteristics and material type of the pin and surrounding tissue necrosis^9^. The alteration and surface treatment of medical implants *via* various chemical and physical techniques is one technique that may improve surface properties, in order to reduce the rate of infection associated with the use of pins associated with fixation devices^10^.

The most commonly isolated bacteria from pin tract infections are opportunistic pathogens. Opportunistic pathogens are often part of the hosts commensal flora which is commonly populated with Gram-positive organisms, with predominating bacterial species including *Staphylococcus* spp., *Micrococcus* spp., and *Corynebacteria* spp.^11^. The bacterial strains selected for this study were *Staphylococcus aureus* and *Staphylococcus epidermidis* since they are the most commonly isolated bacterial strains from infected biomedical devices^12^. *Staphylococcus aureus* infections have been reported to be the most common causative agent of metal biomaterial associated infections, whilst *Staphylococcus epidermidis* are more frequently associated with polymeric biomaterial infections^13^.

The surface coatings selected for use throughout this study were made from zirconium nitride (Ti-ZrNAg) and zirconium nitride containing different concentrations of silver (Ti-ZrN/Ag). Zirconium nitride has been recognised as a potential biomaterial due to its excellent resistance to corrosion, good chemical stability and biocompatibility, including low cytotoxicity to human cells^14,15^. In previous studies, the antimicrobial activity of zirconium nitride-silver, chromium nitride-silver and titanium nitride-silver were investigated. The results suggested that zirconium nitride-silver displayed the most efficacious antimicrobial properties in relation to its tribological properties^15–17^.

The antimicrobial properties of silver have long been established, with silver more commonly used in ion and salt forms with examples being silver sulfadiazine, silver nitrate and as metallic incorporation into medical and health products (such as wound dressings)^18,19^. Silver in its bulk form is inert and has no antimicrobial properties. In order to become antimicrobially active, it requires moisture (such as bodily fluids/water) to ionise the metallic silver (Ag^0^) into three possible oxidation states: Ag^+^, Ag^2+^ and Ag^3+^, of which Ag^+^ is the most common oxidation state^20^. The antimicrobial mechanism of silver is dependent upon its structure (*i.e*. ions, salt, nanoparticles), and in regards to silver ions, the antimicrobial mechanism is closely associated with its interaction with functional groups (*e.g*. thiol groups) in enzymes and proteins^21,22^. This interaction can lead to the disruption and displacement of essential metals in bacterial enzymes, leading to an antimicrobial response due to damaged protein synthesis, DNA and loss of cell membrane integrity, ultimately leading to the death of the bacterial cell^23^. A fundamental study has been previously carried out to determine the effect of the surface properties of ZrN/Ag surfaces on bacterial distribution, clumping and dispersion and it was demonstrated that *S. aureus* was influenced marginally more by surface chemistry whilst *S. epidermidis* cells was influenced marginally more by surface topography^17^.

The aim of this study was to evaluate the antimicrobial efficacy of the Ti-ZrN/Ag coatings against *S. aureus* and *S. epidermidis*, focusing on contact kill mechanisms and the respiration status of the microorganisms. Furthermore, the cytotoxicity of the TiZrN coatings, against a human cell line were also elucidated.

## Results

### Chemical composition

**Figure 1:**
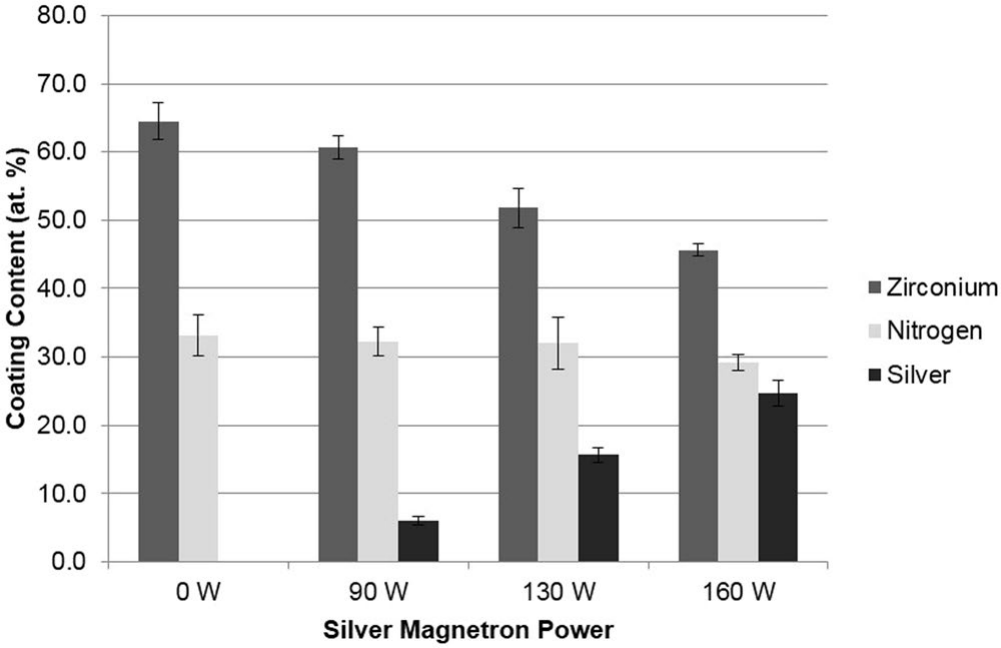
EDX analysis of the four varying coating compositions, displaying the zirconium, nitrogen and silver content in relation to magnetron power.

**Figure 2:**
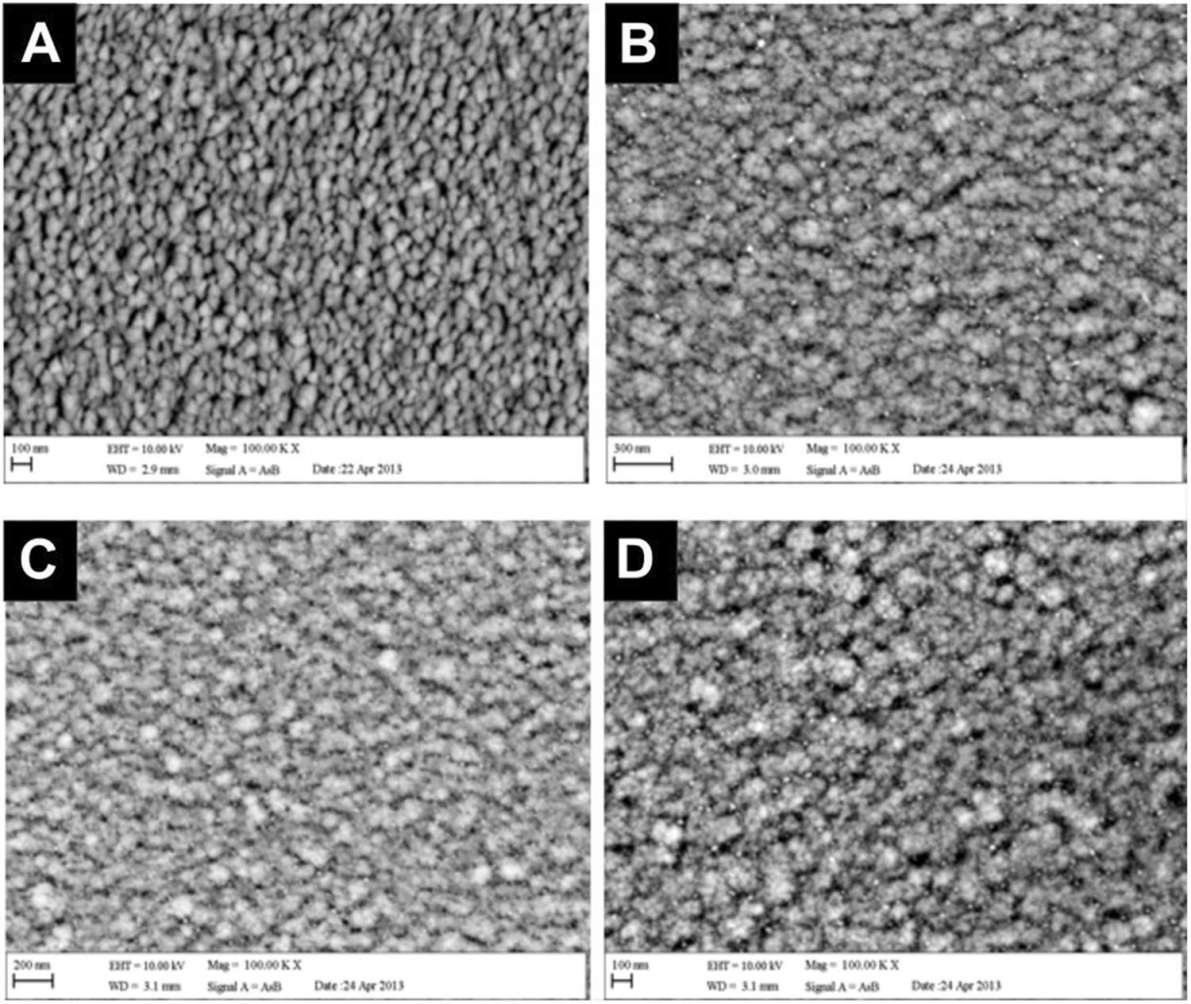
Electron backscatter detector images of the four coatings (X 100,000 magnification); (**A**) Ti-ZrN, (**B**) Ti-ZrN/6.0 at% Ag, (**C**) Ti-ZrN/15.6 at% Ag and (**D**) Ti-ZrN/24.7 at% Ag. The white particles seen on **(B**), (**C**) and (**D**) are silver nanoparticles. No particle appears to be any larger than 10 nm in (**B** and **C**) but the particles are larger on d (10–20 nm).

Energy Dispersive X-Ray (EDX) analysis was undertaken on the four Ti-ZrN/Ag coatings. The silver content demonstrated averages of 6.0%, 15.6% and 24.7% for magnetron powers of 90 W, 130 W and 160 W respectively (Fig. 1). Backscattered electron images were obtained using the backscattered electron detector, which resulted in visualisation of the chemical elemental distribution on the surface. Due to heavier elements being able to backscatter electrons more freely, they appeared on the image as a brighter phase resulting in the silver (heavier than zirconium) to be visualised as white nanoparticles thus showing their distribution throughout the surfaces (Fig. 2). The Ti-ZrN was examined and characterised as a negative control, which showed a relatively homogenous surface, demonstrated by a similar colour phase (Fig. 2A). The Ti-ZrN/6.0 at% Ag showed silver particles of various sizes around 8 nm, with large variations being observed in their distribution (Fig. 2B). The Ti-ZrN/15.6 at% Ag displayed a different morphology, with the overall size of the silver nanoparticles being around 5 nm and these were more regularly distributed but more tightly packed (Fig. 2C). The Ti-ZrN/24.7 at% showed mixed sized, silver particles (ranging from 10–20 nm), which were closely spaced (Fig. 2D).

### Structural Morphology

**Figure 3:**
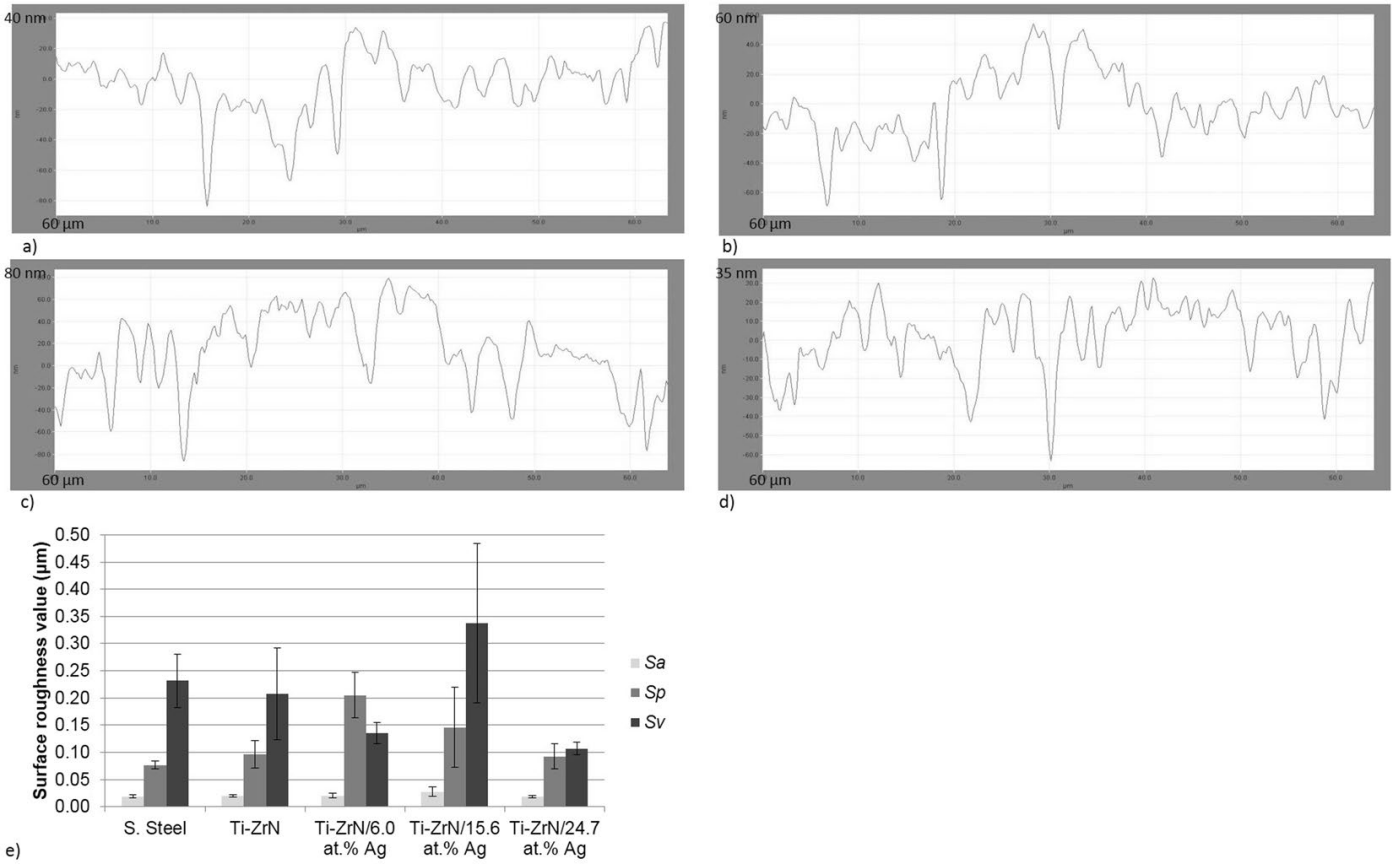
Line profiles of the surface coatings visualised *via* optical profilometry (**a**) Ti-ZrN, (**b**) Ti-ZrN/6.0% Ag, (**c**) Ti-ZrN/15.6% Ag and (**d**) Ti-ZrN/24.7% Ag, demonstrating the differences in microtopography of the four coatings. Note the Y axis scale differs for each image (**e**) shows the *S*_*a*_, *S*_*p*_ and *S*_*v*_ values.

The surface microtopography was determined to visualise and quantify surface features that were of microbial dimensions. Results from the linear profiles demonstrated that the Ti-ZrN coatings did not alter surface microtopography, even in the presence of an increased silver concentration. The average width of the features from the linear profiles ranged between 2.9 µm (Ti-ZrN) and 2.0 µm (Ti-ZrN/6.0 at% Ag) (Fig. 3a–d) and were demonstrated to be larger than the average of a size of a *Staphylococcus* spp. cell (1 µm). However, the average depth values were in the range of 0.029 µm–0.037 µm and therefore were not significant in relation to the size of a *Staphylococcus* spp. cell (Figs 3a–d).

The white light profilometer was used to obtain roughness values of the surfaces at a microtopography scale. Of the five surfaces, no significant difference (*p* > 0.05) was demonstrated in the *S*_*a*_ values (arithmetic average height) (0.02–0.03 µm) although for the silver containing surfaces, the *S*_*a*_ values demonstrated the same trend as the *S*_*v*_ values (surface valley depth) (Fig. 3e). *S*_*p*_ values, which represent the surface peak height showed that the lowest value recorded was on the stainless steel control and the pure Ti-ZrN (0.08 µm and 0.10 μm, respectively), whilst the silver coatings decreased from the greatest *S*_*p*_ value (0.21 µm) for the Ti-ZrN/6.0 at% Ag coating and decreased with increasing silver content to 0.09 µm for the Ti-ZrN/24.7 at% Ag coating (Fig. 3e). The *S*_*v*_ values (valley depths), demonstrated that the stainless steel and Ti-ZrN coatings were similar (0.23 µm and 0.21 µm respectively), whilst although lower the Ti-ZrN/6.0 at% Ag and Ti-ZrN/24.7 at% Ag coatings also demonstrated similar values (0.14 µm and 0.11 µm respectively), whilst the Ti-ZrN/15.6 at% Ag) demonstrated the greatest *S*_*v*_ value (0.34 µm) (Fig. 3e).

**Figure 4:**
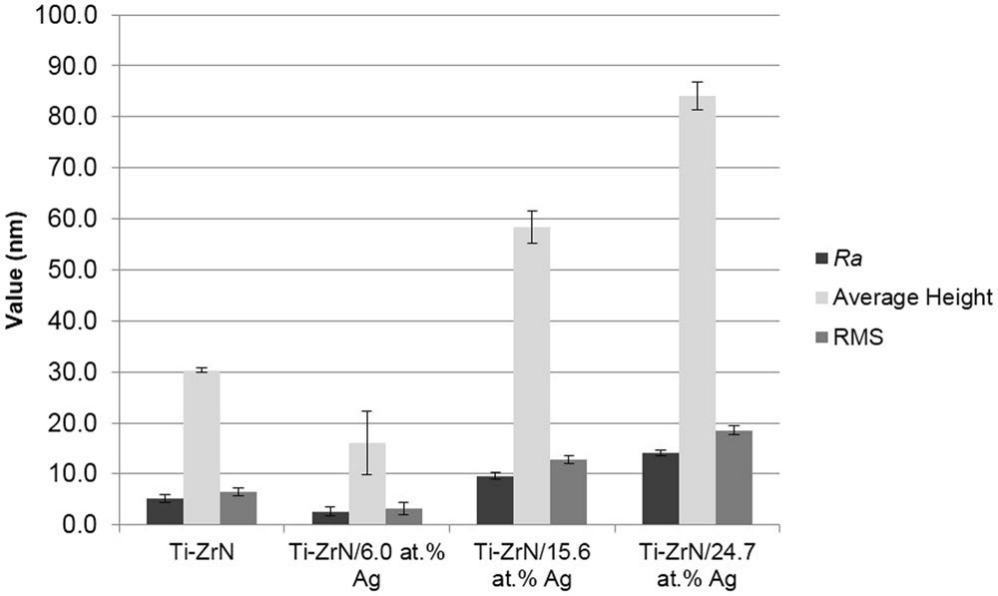
AFM values of the four coatings (**a**) Ti-ZrN, (**b**) Ti-ZrN/6.0 at% Ag, (**c**) Ti-ZrN/15.6 at% Ag and (**d**) Ti-ZrN/24.7 at% Ag, demonstrating the differing nanotopographies of the four surfaces.

The AFM results (Fig. 4) demonstrated that on the surfaces containing silver, the *R*_*a*_ (Arithmetic average height) (2.6–14.1 nm), average height (16.2–84.1 nm) and RMS (Root mean square roughness) values (3.2–18.6 nm) all increased with increasing silver content on the Ti-ZrN/6.0 at% Ag, Ti-ZrN/15.6% Ag and Ti-ZrN/24.7 at% Ag surfaces respectively.

**Figure 5:**
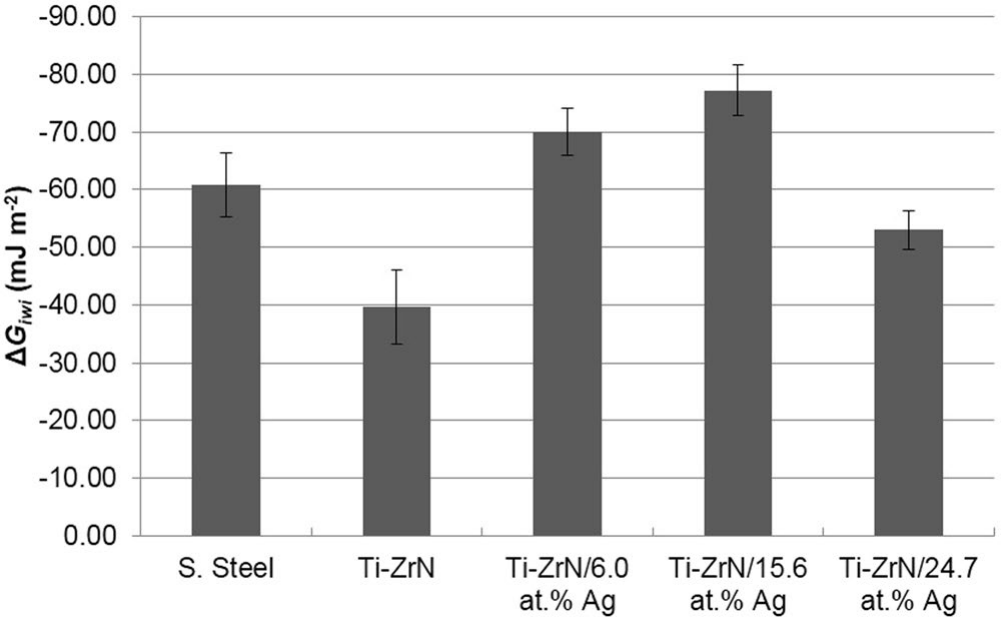
Hydrophobicity testing of the surfaces whereby the ∆*G*_*iwi*_ of the samples were all negative scale, demonstrating hydrophobic surfaces.

The hydrophobicity of the coatings were calculated by obtaining contact angles using two polar solvents (water and formamide) and one non-polar solvent (1-bromonaphthalene), in order to calculate the *Δ*G_*iwi*_, (the quantitative measure for hydrophobicity/hydrophilicity). The results demonstrated that all five surfaces tested possessed negative *Δ*G_*iwi*_ values indicating a hydrophobic nature. The pure Ti-ZrN was the least hydrophobic at *Δ*G_*iwi*_ −40, whilst the Ti-ZrN/15.6% Ag was the most hydrophobic at *Δ*G_*iwi*_ −77 (Fig. 5; Table 1). All the surfaces were significantly different to one another, with the exception being between the 6.0% Ag and 15.6% Ag coatings (Fig. 5; Table 1).

**Table 1:** Surface tension parameters for polar and apolar liquids, used to calculate physicochemical parameters (Oss *et al*., 1990)^59^.

Liquid	γ_L_ mJ m^−2^	γ_L_^LW^ mJ m^−2^	γ_L_^+^ mJ m^−2^	γ_L_^-^ mJ m^−2^
Water	72.8	21.8	25.5	25.5
Formamide	58	39	2.28	39.6
*α-*Bromonapthhalene	44.4	44.4	—	—

**Figure 6:**
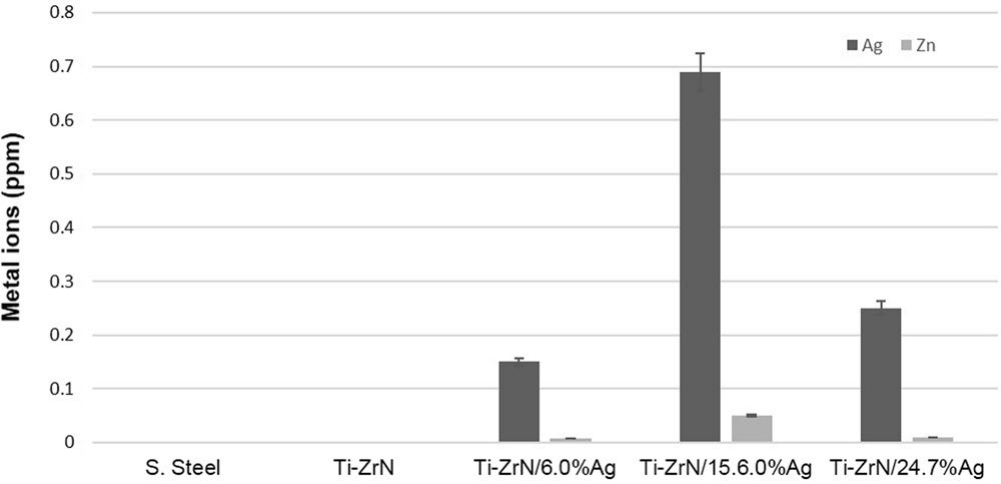
Ion coupled plasma – atomic emission spectroscopy (ICP-AES) results of the silver and zirconium release from the coatings following 24 h incubation at 37 °C.

Ion coupled plasma –atomic emission spectroscopy (ICP-AES) was used to quantify leaching of elements from the coatings (silver and zirconium). The results demonstrated that the silver coatings leached silver (Fig. 6) into the surrounding broth with an increase in silver ions leached with increasing silver concentration. After 24 h, the number of silver ions leached from the silver containing surfaces was 0.15 ppm, 0.69 ppm and 0.25 ppm (Ti-ZrN/6.0 at% Ag, Ti-ZrN/15.6 at% Ag and Ti-ZrN 24.7 at% Ag respectively).

All of the surfaces tested demonstrated extremely low zirconium leaching (Fig. 6) with similar values following 24 h, Ti-ZrN/6.0 at% Ag 0.008 ppm; Ti-ZrN/15.6 at% Ag 0.05 ppm and Ti-ZrN 24.7 at% 0.009 ppm.

### Antimicrobial analysis

**Figure 7:**
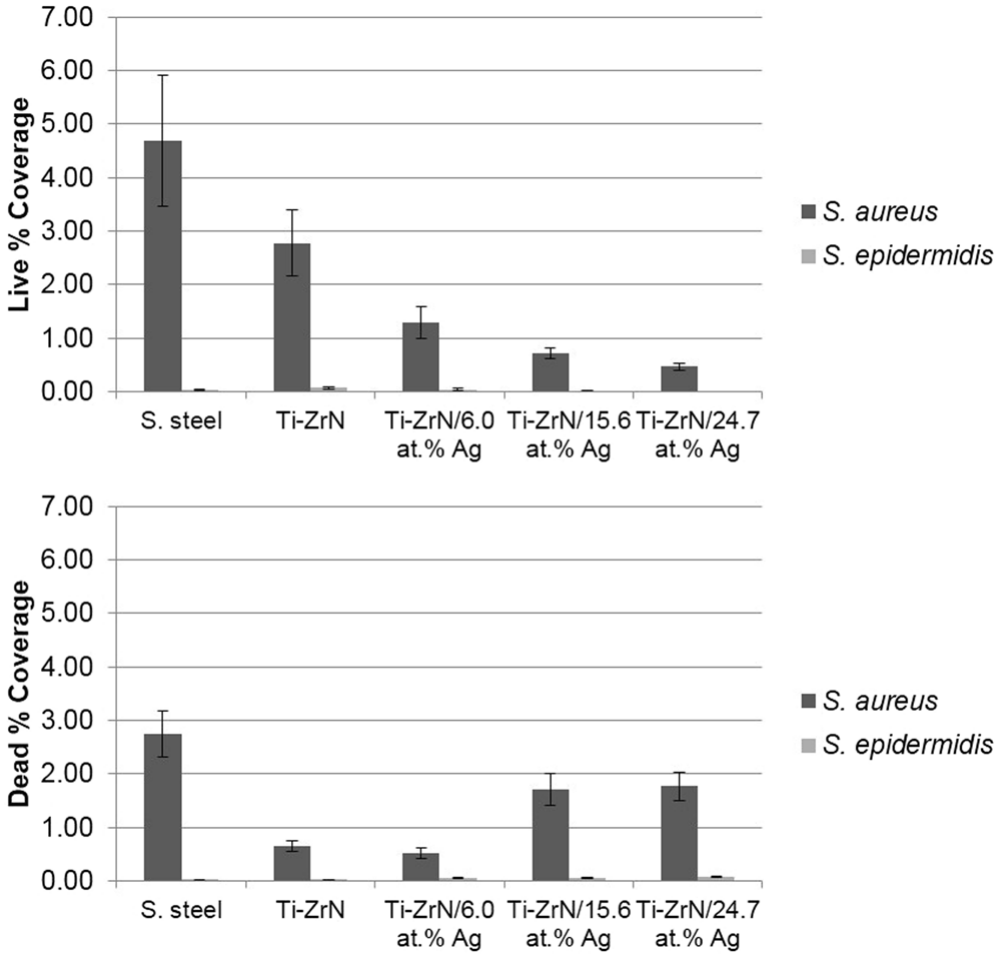
LiveDead™ staining of the five surfaces (including the stainless steel control) against *S. aureus* and *S. epidermidis*, with (**a**) showing live% coverage and (**b**) dead% coverage.

In order to determine the viability of the microorganisms retained, LiveDead™ staining was used (Fig. 7). Overall the numbers of live (Fig. 7a) and dead cells (Fig. 7b) retained were greatest on the stainless steel surfaces. Both the numbers of live and dead *S. epidermidis* cells were retained in fewer numbers (*S. epidermidis* percentage coverage range live 0.00–0.07%; dead 0.02–0.07%) than *S. aureus* (percentage coverage range 0.47–4.69%; dead 0.65–2.75%) (Fig. 7a). The results demonstrated that the Ti-ZrN and Ti-ZrN/Ag surfaces contained less viable *S. aureus* and *S. epidermidis* cells on the surface than the stainless steel control, and a decrease in live cells was observed as the silver content increased (live cells *S. aureus* 2.78%, 1.30%, 0.72%, 0.47% and *S. epidermidis* 0.07%, 0.04%. 0.01% and 0.00% Ti-ZrN, Ti-ZrN/6.0 at% Ag, Ti-ZrN/15.6 at% Ag and Ti-ZrN/24.7 at% Ag respectively) (Fig. 7a). On the silver containing surfaces the percentage coverage of the retained live bacteria followed the same trend as the surface roughness *S*_*p*_ values. The Ti-ZrN/15.6at.Ag (1.71%) and Ti-ZrN 24.7 at% (1.77%) surfaces demonstrated a significant increase in dead *S. aureus* cells compared to the Ti-ZrN (0.65%) and Ti-ZrN/6.0 at% Ag (0.52%) coatings, however there was no significant increase in dead cells between the Ti-ZrN/15.6 at% Ag and the Ti-ZrN/24.7 at% Ag surfaces. The number of dead *S. epidermidis* cells (Fig. 7b) was found to be greatest on the Ti-ZrNAg containing surfaces at 0.03%, 0.05%, 0.06% and 0.07% on the Ti-ZrN, Ti-ZrN/6.0 at% Ag, Ti-ZrN/15.6 at% Ag and Ti-ZrN 24.7 at% Ag respectively.

**Figure 8:**
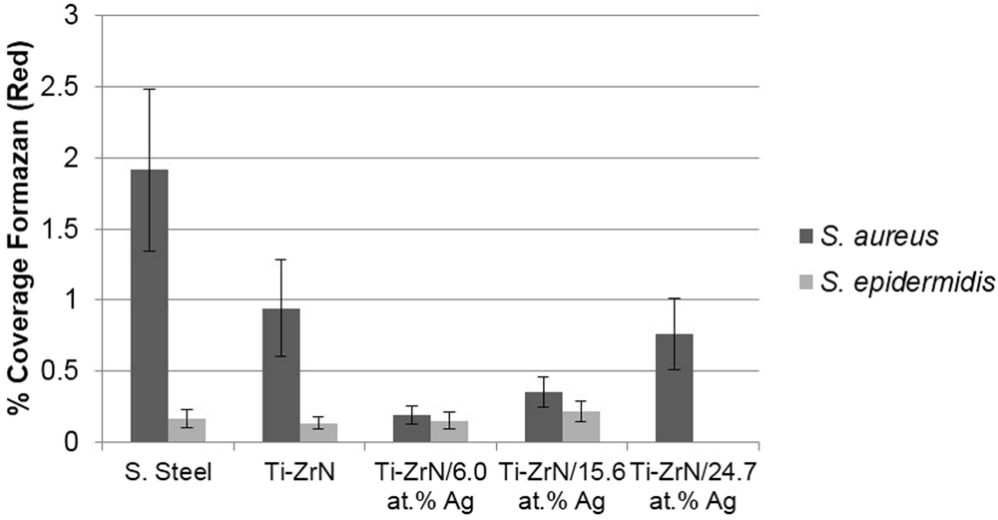
CTC-DAPI staining indicating the percentage coverage of respiring bacteria on the surfaces.

CTC-DAPI fluorescent staining demonstrated the metabolic activity of the bacteria and the counterstain of the total bacterial cells (Fig. 8). The results demonstrated that when compared to the stainless steel of Ti-ZrN surfaces, the silver present reduced the coverage of respiring *S. aureus* cells but an increase in silver content increased the amount of respiring bacteria (0.94%, 0.20%, 0.35% and 0.76% on the Ti-ZrN, Ti-ZrN/6.0 at% Ag, Ti-ZrN/15.6 at% Ag and Ti-ZrN/24.7 at% Ag surfaces respectively. The results obtained from the *S. epidermidis* showed similar percentage coverage on each surface (range 0.14–0.22%), with an exception being the Ti-ZrN/24.7 at% Ag where no viable bacteria were observed.

### Cytotoxicity Assay

**Figure 9:**
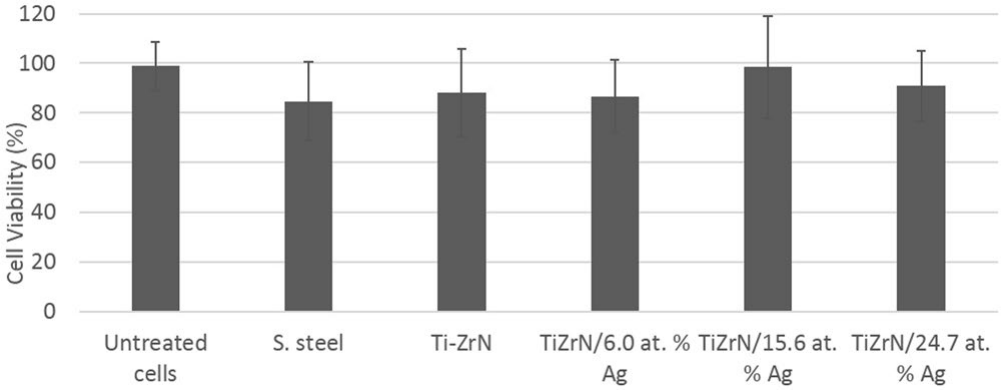
Cytotoxicity results of the five surfaces against a human monocyte cell line (U937). Results show no significant difference (*P* < 0.05) when compared to the untreated cells (negative control), indicating no cytotoxic effect was exhibited by these surfaces towards this specific cell line.

In order to assess the surface coatings suitability for application in conjunction with biomaterials, such as pin tract infections cytotoxicity assays were carried out (Fig. 9). The Ti-ZrN and Ti-ZrNAg surface coatings were tested against a commercial human monocyte cell line U937 utilising a cell viability (MTS) method. Results demonstrated that all of the surfaces demonstrated little to no cytotoxic effect. The negative control (untreated cells) gave the highest percentage viability (98.95%), followed by TiZrN/15.6 at% Ag (98.54%). The increase in silver concentration had no detrimental effect as TiZrN/24.7 at% Ag produced a higher percentage viability than TiZrN/6.0 at% Ag, producing values of 90.78% and 86.72%, respectively. Stainless steel was the most detrimental surface producing a percentage viability of 84.69%. Further, the results of the coated surfaces showed no significant difference (*P* < 0.05) when compared to the untreated cells (negative control).

## Discussion

A range of surface coatings were produced on medical grade stainless steel in order to determine if the number of viable bacteria retained could be reduced. EDX analysis showed that the silver contents of the coatings were Ti-ZrN/6.0 at% Ag, Ti-ZrN/15.6 at% Ag and Ti-ZrN/24.7 at% Ag silver for magnetron powers of 90 W, 130 W and 160 W, respectively. All of the coatings had a stoichiometric zirconium and nitrogen ratio. The nitrogen content appeared to be lower than expected, however this could be due to nitrogen’s low molecular weight making it difficult to detect using EDX.

Backscattered electron detection demonstrated the distribution of silver particles throughout the surface coatings. The silver particles were situated close together, thus allowing antimicrobial activity if bacteria were to make contact with the surface (*via* contact with the silver particle). Throughout this study, a higher silver concentration correlated with a higher the antimicrobial efficacy (as observed in live percentage coverage). This contact-initiated mode of antimicrobial action is well established in the case of silver nanoparticles, which provide a much greater degree of access due to silver ions due to their large surface area^20^. Upon contact with the bacteria, the silver nanoparticles may leach ions which attach to the cell membrane and penetrate the bacterial cell^20^, interact with sulphur-containing proteins (as well as phosphorous containing compounds such as DNA), and attach to thiol groups^24,25^. Thus, silver has shown to inhibit respiration and growth, by damaging protein and enzymes, ultimately leading to loss of cell membrane integrity and cell death^20,26–28^. Previous studies into nitride/silver nanocomposites have demonstrated that not all microorganisms are susceptible to leaching of silver, whereas composite-coatings have been suggested to have a contact kill efficacy^16,29^.

The microtopography of a substrate is a vital parameter when evaluating antimicrobial efficacy, specifically when surfaces have features around the micron range, which is similar to that of the dimension of a bacterium (in this case *Staphylococci*), which can result in an increase in bacterial retention^30,31^. The optical profilometry line profiles, demonstrated that the deposition of thin films (1 µm thick) onto stainless steel using magnetron sputtering did not affect the microtopography of the surfaces. This was confirmed *via S*_*a*_ values which were similar for all surfaces indicating that the coatings did not affect the micro-roughness of the surfaces. *S*_*v*_ demonstrated the same trend as the *S*_*a*_ values which also was the same trend observed of the formazan production of *S. epidermidis* in the CTC-DAPI assays. However, the *S*_*p*_ value demonstrated the same decreasing trend seen on the silver surfaces of the number of the live cells of both *S. aureus* and *S. epidermidis* suggesting that as the concentration of silver increases, so do the tops of the nanoparticles that protruded from the matrix of the Ti-ZrNAg which results in a greater antimicrobial kill. This was confirmed by the AFM values which demonstrated that with increasing silver content, the nanotopography of the surfaces also increased.

Hydrophobicity varied throughout the surface coatings and with the silver containing coatings, surface hydrophobicity was found to follow the same trend as the *S*_*a*_ and *S*_*v*_ surface results. All surfaces exhibited hydrophobicity, with Ti-ZrN/15.6 at% Ag the most hydrophobic (∆*G*_*iwi*_ −77.22) and Ti-ZrN the least (∆*G*_*iwi*_ −39.63)^32^.

Zirconium and silver ion leaching was analysed *via* ICP-AES. In the case of silver, leaching of the ions into the surrounding broth was observed. The minimum inhibitory concentration (MIC) of silver against *Staphylococci* spp., has been demonstrated to be around 10 ppm^33^. Sandstrom (2011) found that silver produced an MIC of 8 ppm towards *S. aureus* and a minimum bactericidal concentration (MBC) of 32 ppm^34^. However, in our work, the leaching of the silver ions into the broth was much lower than the bacteriostatic value obtained for the MIC. This may be a result of the bacteria being in contact with the silver on the surfaces, since work by Vaidya *et al*., (2017) demonstrated that silver in ionic form did not demonstrate high levels of antimicrobial activity when compared to other metal ions^35^. In the case of zirconium ions, all the surfaces demonstrated extremely low levels of leaching. In a previous study, it had been established that this level of leaching would not be enough to damage human cells – with a toxic amount being around 150 ppm^36^.

Cytotoxicity results demonstrated that the Ti-ZrN and Ti-ZrNAg surface coatings had no detrimental effect upon the human monocyte cell line U937. Zirconium is already widely used in the construction of prosthetic devices, due to its excellent biocompatibility to human cells and good mechanical and chemical properties^37–39^. Further, when exposed to oxygen, zirconium becomes zirconium oxide which is also biocompatible, thus making it an ideal material for application within pin tracts^40^. The oligodynamic characteristics of silver are well documented throughout the literature^19,41,42^. This, coupled with good biocompatibility has led to silver being used in a plethora of biomedical applications including, wound dressings^43–45^, antibacterial cream for burns patients (silver sulfadiazine)^46,47^ and surface coatings for equipment/in-dwelling patient devices^48–50^. In light of the cytotoxicity results, the application of TiZrN in combination with silver for pin tracts, has promising potential to reduce bacterial colonisation, without possessing a detrimental effect to the host cells.

LiveDead™ staining results demonstrated a decrease in live *S. aureus* cells on the silver containing coated samples, and an increase in antimicrobial efficacy (reduction of live cells) as silver concentration increased. An increase in dead percentage coverage was observed against the coatings with a higher silver concentration with TiZr-N/15.6 at% Ag and TiZr-N/24.7 at% Ag. For *S. epidermidi*s, *an* extremely low percentage coverage of live and dead cells were observed on all the substrata. A decrease in the percentage coverage of live *S. epidermidis* cells was also observed with increasing silver concentration. The LiveDead™ staining kit, stains all bacterial cells green using CYTO-9, whilst cells with loss of membrane integrity are the over-stained using propidium iodide (red)^51^. However, cells may still be viable with a compromised membrane, therefore other methods should be used to check cell viability, especially if silver ions have the ability to enter the cell *via* channels, leading to DNA damage and growth inhibition^52,53^.

The CTC-DAPI staining assay, displayed the respiration of individual microorganisms, due to the reduction of tetrazolium (in the electron transport chain of the microorganism), leading to the production of insoluble, fluorescent formazan^54–56^. Results demonstrated that *S. aureus* displayed more respiring cell coverage than *S. epidermidis*. It was demonstrated that an increase in silver content did not decrease cell respiration as expected, but there was a significant increase in *S. aureus* respiration in comparison to the stainless steel control. *S. epidermidis* resulted in similar respiration on all the surfaces with the exception of the Ti-ZrN/24.7 at% Ag suggesting that the silver ions affect the bacteria in different ways^57,58^. Growth inhibition from bacteriostatic antibiotics have been shown to supress cellular respiration, whilst bactericidal antimicrobial formations are associated with accelerated respiration^50^. Therefore, it might be suggested that more than one assay should be used in order to confirm cell viability.

## Conclusion

The Ti-ZrN/Ag coatings demonstrated antimicrobial efficacy. Surface hydrophobicity followed the same trend as the *S*_*a*_ and *S*_*v*_ values. *S*_*p*_ values demonstrated the same trend as the increase in silver concentration in the surfaces since the silver produced nano-peaks. The changes in nanotopography with increasing silver concentration was confirmed with AFM. The increase in the silver concentrations of the surfaces resulted in a decrease in bacterial viability. Cytotoxicity testing revealed the Ti-ZrN/Ag coatings elicited no detrimental effect against a human monocyte cell line. This work demonstrated that such coatings have the potential to reduce the viability of bacteria that result in pin tract infections.

## Materials and Methods

### Bacterial Preparation

The microorganisms *Staphylococcus aureus* NCTC 8532 and *Staphylococcus epidermidis* NCTC 11047 were used throughout this study. Stock cultures were stored at −80 °C. When required the cultures were thawed and inoculated onto nutrient agar media (Oxoid, UK) and incubated at 37 °C for 24 h. The inoculated agar plates were kept refrigerated at 4 °C and replaced every four weeks to maintain the genotype. Sterile brain heart infusion (BHI) broths (Oxoid, UK) (10 mL) were inoculated with *S. aureus* or *S. epidermidis* and incubated overnight in an orbital incubator (610* g*) at 37 °C for 24 h. Cultures were removed from incubation and the cells were washed in sterile membrane filtered water (MilliporeElix, USA) (10 mL) by centrifuging at 600 g for 8 min. The supernatant was removed and the cells were re-suspended in sterile distilled water. Cells were diluted to an optical density (OD) of 1.0 ± 0.05 at 540 nm using a spectrophotometer (Jenway 6305, UK), calibrated against distilled water. Cell numbers were determined in colony forming units/mL (CFU/mL) using serial dilutions and were determined to be 0.33 × 10^8^ CFU/mL for *S. aureus* and 0.76 × 10^8^ CFU/mL for *S. epidermidis*. The diluted cell suspension (100 µL) was spread and repeated in duplicates on brain heart infusion agar (Oxoid, UK) and incubated at 37 °C for 24 h.

### Surface coatings

The surface coatings were deposited onto medical grade 316 L stainless steel (Aalco, UK), at with a range of different powers used on the silver target in order to produce a range of silver concentrations in the coatings (90 W, 130 W and 160 W). The Ti in the surface coating title denotes that a titanium interlayer was used to improve the adhesion of the coating to the surface of the stainless steel, however the titanium was not evident as part of the chemistry of the surface coatings.

### Scanning Electron Microscopy (SEM), Energy-Dispersive X-ray (EDX) Spectroscopy and Electron Backscattering analysis

Scanning electron microscopy was used to display the coatings morphology and structure using a Zeiss Supra VP40 field emission gun scanning electron microscope. EDX was performed on the samples to determine the chemical composition of the coatings (Edax Trident) using an EDAX Sapphire Si (Li) detector, and quantified using a standardless ZAF algorithm. The chemical composition was calculated as an atomic percentage (at%), giving the percentage of the said atom relative to the total number of atoms in the scan. Electron backscattered imaging spectroscopy was undertaken on the samples to map the silver particle distribution on the substrate (n = 3).

### Atomic Force Microscopy

Roughness parameters were obtained using an explorer AFM (Veeco, USA) operated in contact mode using a force constant of 0.12 Nm^−1^ and a silicon nitride tip. Scans with the Ti-ZrN coating and Ti-ZrN/Ag coatings were performed. Three replicate scans were performed on three separate coupons (n = 3).

### Hydrophobicity Measurements

The hydrophobicity was calculated by obtaining the surface contact angles of two polar liquids: HPLC grade water (Fisher Scientific; Loughborough, UK) and Formamide (Sigma Aldrich, UK) and one non-polar solvent: 1-bromonaphthelene (Sigma Aldrich, UK). The contact angles were obtained using the sessile drop technique (Kruss MobileDrop II, Kruss, Germany). This instrument deposited 5 μL volume drops from a standard dropper. The drop contact angle on the surface is then measured using a camera and prism inside the device. The image was analysed using Kruss SW23 (DSA2) (Kruss, Germany) analysis software, using the Young-Laplace bubble fit method to obtain the angle between the ‘bubble’ and the surface interface. Different surfaces were used for each solvent. Surface tension parameters for polar and apolar liquids were used to calculate physicochemical parameters (Table 1) (Oss *et al*. 1990)^59^ (n = 5).

### Surface free energies and components

Determination of the surface free energy and components were determined using the van Oss and Good (van Oss *et al*., 1986; van Oss, 1995) calculation^60,61^. The surface free energy (γ_s_) can be determined from the apolar Lifshitz –van der Waal component (γ_s_^LW^) and the polar or Lewis acid base component (γ_s_^AB^) where^60^;

1 $${{\rm{\gamma }}}_{{\rm{s}}}={{\rm{\gamma }}}_{{\rm{s}}}^{{\rm{LW}}}+{{\rm{\gamma }}}_{{\rm{s}}}^{{\rm{AB}}}$$

The polar component comprises of two parameters^60^;

2 $${{\rm{\gamma }}}_{{\rm{s}}}^{{\rm{AB}}}=2\surd {{\rm{\gamma }}}_{{\rm{s}}}^{+}{{\rm{\gamma }}}_{{\rm{s}}}^{-}$$

where γs^+^ is the electron acceptor and γ_s_^−^ the electron donor of the polar surface tension component. The apolar components of the surface and liquid are combined into (van Oss 1995)

3 $${{\rm{\gamma }}}_{{\rm{sL}}}^{{\rm{LW}}}={(\surd {{\rm{\gamma }}}_{{\rm{s}}}^{{\rm{LW}}}-\surd {{\rm{\gamma }}}_{{\rm{L}}}^{{\rm{LW}}})}^{{\rm{2}}}$$

whilst for the polar components (van Oss 1995)^60^;

4 $${{\rm{\gamma }}}_{{\rm{sL}}}^{{\rm{AB}}}={\rm{2}}(\surd {{\rm{\gamma }}}_{{\rm{s}}}^{+}{{\rm{\gamma }}}_{{\rm{s}}}^{-}+\surd {{\rm{\gamma }}}_{{\rm{L}}}^{+}{{\rm{\gamma }}}_{{\rm{L}}}^{-}-\surd {{\rm{\gamma }}}_{{\rm{s}}}^{+}{{\rm{\gamma }}}_{{\rm{L}}}^{-}-\surd {{\rm{\gamma }}}_{{\rm{s}}}^{-}{{\rm{\gamma }}}_{{\rm{L}}}^{+})$$

where s stands for solid, and L stands for the contact liquid.

By combining equations 3 and 4, the surface free energy values can be calculated (van Oss *et al*. 1986)^61^;

5 $${{\rm{\gamma }}}_{{\rm{L}}}({\rm{1}}+\,\cos \,\theta )=2(\surd {{\rm{\gamma }}}_{{\rm{s}}}^{{\rm{LW}}}{{\rm{\gamma }}}_{{\rm{L}}}^{{\rm{LW}}}+\surd {{\rm{\gamma }}}_{{\rm{s}}}^{+}{{\rm{\gamma }}}_{{\rm{L}}}^{-}+\surd {{\rm{\gamma }}}_{{\rm{s}}}^{-}{{\rm{\gamma }}}_{{\rm{L}}}^{+})$$

### Hydrophobicity measurements of surfaces

Surface hydrophobicity was calculated using the free energy of interaction (Δ*G*_*iwi*_)

6 $${{\rm{\Delta }}G}_{iwi}=-\,{2{\rm{\gamma }}}_{{\rm{sL}}}$$

### Ion coupled plasma –atomic emission spectroscopy (ICP-AES) Analysis

ICP-AES was used to evaluate the release of silver ions as well as the release of zirconium ions, into a liquid medium. The coupons were cleaned using ethanol and distilled water and then put inside a sealed beaker and autoclaved at 121 °C for 15 min. Sterile brain heart infusion broths (100 mL) (Oxoid, UK) were used for the liquid medium to replicate a nutrient rich substance. The coated stainless steel coupons (n = 16) were added to the broth aseptically and sealed with a sterile foam bung and aluminium foil. The samples were placed in an orbital incubator at 37 °C at 150 RPM. After of 24 h, 10 mL samples were extracted aseptically and diluted 1:1 in HPLC Grade Water (Fisher Scientific, UK) and filter sterilised with a 0.2 μm filter membrane (Acrodisk, UK) and a Luerlock™ syringe (Sigma Aldrich, Dorset, UK). Samples were stored at 4 °C in the dark until analysis was to be undertaken. ICP-AES was performed on Varian Vista XA (CCD simultaneous ICP-AES) with sample introductory system of glass spray chamber and glass nebuliser. Analysis was undertaken using blanks of the brain heart infusion broth as a negative control and liquid standards of silver and zirconium (Sigma Aldrich, Dorset, UK) were used in the ICP instrument to produce a calibration curve of 0.1, 0.5, 1.0 and 5.0 parts per million (ppm) concentrations.

### White Light Profilometry

Five topography, and images of each surface three replicates of each surface were taken (n = 15). Images were taken using a MicroXAM (phase shift) surface mapping microscope on the highest magnification setting (x101.61 magnification) (Omniscan, UK). Analysis was carried out using EX mode. The image analysis software used was Mapview AE 2Æ17 (Z range 210.5–1.169 μm) (Omniscan, UK).

### LIVE/DEAD™ Staining

To monitor cell viability, cells retained on surfaces were treated using a live/dead stain (LIVE/DEAD™ Baclight™ bacteria viability kit, Invitrogen, Scotland). After diluting the stains in dimethyl sulfoxide (Sigma, UK) according to the manufacturer’s instructions, the propidium iodide and Syto 9 dye components were individually diluted in a 1:10 solution in sterile distilled water in eppendorf tubes. Five microliters of each dilution was then mixed together, and 10 μL was spread across the sample and allowed to air dry in the dark in a microbiological Class II safety hood. Following drying samples were stored in the dark at 4 °C. Potentially viable and damaged cells were distinguished under the fluorescence microscope (Nikon Eclipse E600, Surrey, UK) since it is assumed that viable cells appeared green, whilst non-viable or membrane compromised cells appeared red. The microscope was mounted with an F-View II black and white digital camera (Soft Imaging System Ltd., Helperby, UK, supplied by Olympus, Hertfordshire, UK). This system used a Cell F Image Analysis package (Olympus, Hertfordshire, UK). The percentage coverage of the live and dead cells retained on the surface was measured using separate selective UV filters across the same field of view. Twenty fields of view using each UV filter were taken per surface.

### CTC DAPI Assays

5-cyano-2,3-ditolyl tetrazolium chloride (CTC) is a redox dye which reduces into formazan and fluoresces red in the presence of UV radiation. Coupons were subjected to a standardised retention assay before staining^17^. CTC (Sigma Aldrich, UK) was prepared from powder and dissolved to a 5 mM concentration in sterile membrane filtered water (Millipore Elix, Germany), the CTC was also filter sterilised (0.2 μm Acrodisk, UK) before use.

Direct staining of the coupons was performed by pipetting 500 μL of CTC onto the substrata/retained bacteria and incubated in a sealed container, without agitation, for 30 min at 37 °C. Following incubation, the surfaces were rinsed with sterile distilled water with a 3 mm nozzle at a 45° angle for five seconds and counter stained by pipetting 500 μL of 1 μg/mL 4′,6-diamidino-2-phenylindole (DAPI) (Sigma Aldrich, UK), prepared from the powder dissolved in sterile membrane filtered water. This was then pipetted onto the surface and left to incubate at room temperature in the dark, in a class II microbiological flow cabinet for 15 min. The coupons were rinsed again with sterile distilled water and then left to dry in the class II cabinet for 45 min until the coupon surface was completely dry. Stained samples were stored at 4 °C, in the dark and the substrata plus retained cells were visualised using epifluorescent microscopy (Nikon Eclipse E600 epifluorescence microscope, Tokyo, Japan), and Cell-F image visualisation software (Olympus, UK) was used for image capture and analysis. The two stains were differentiated by using filters of 590/650 nm for red excitation and emission spectra (CTC/Formazan) and 350/470 nm for the blue (DAPI) excitation and emission spectra, respectively.

### Cytotoxicity Assays

#### Cell Culture

The commercial human monocyte cell line U937 (Health Protection Agency Culture Collections, Salisbury, UK) was cultured under aseptic conditions at 37 °C and 5% CO_2_ in RPMI-1640 media (Lonza, Belgium) supplemented with 10% foetal bovine serum (Sigma-Aldrich, Dorset, UK) and 2% penicillin-streptomycin (Lonza, Slough, UK). The U937 cell suspension was maintained at 5 × 10^5^ million cells/mL by resuspension in fresh media every alternate day. Sterile filtered 0.4% trypan blue dye (Sigma-Aldrich, UK) was used to check cell viability using a 1:1 ratio of cell suspension to trypan blue and then counting the number of non-viable cells that take up blue dye using a TC10 automated cell counter (BioRad, Singapore). The viability of cells was above 90% for experimental purposes.

### Cell Viability (MTS)

S. steel, Ti-ZrN, Ti-ZrN/6.0 at% Ag, Ti-ZrN/15.6 at% Ag and Ti-ZrN/24.7 at% were sterilized with IMS before being placed in a 24-well plate. U937 monocytes were seeded in the 24-well plate (5 × 10^5^ cell per well) containing the metals, and incubated at 37 °C and 5% CO_2_ for 24 h. Cells (2.5 × 10^5^ cell) were then spun down at 500 g for 5 minutes and re-suspended in 100 µL RPMI-1640 media. A 20 µL volume of Cell Titer 96 AQueous One Solution Reagent (Promega, UK) was added to the cells and incubated at 37 °C and 5% CO_2_ for 1–4 h. Formazan absorbance was measured at 490 nm and results are expressed as percentage of the untreated control cells. U937 cells seeded in blank wells were used as a negative control. Cells treated with pure ethanol were used as a positive control.

### Statistical analysis

The standard error of the mean was shown on the graphs using error bars. *p* values were calculated at the 95% confidence level using student t-tests. For the cytotoxicity assays, IBM SPSS Statistics (Version 22) was used to perform all statistics. The normality test and one way ANOVA with Tukey pairwise comparisons were conducted on all the data. Graphs were drawn using Microsoft Excel 2013. In all cases, *P* < 0.05 was considered statistically significant.

### Data availability

The datasets generated during and/or analysed during the current study are available from the corresponding author upon reasonable request.

## References

[1] Park, J. *Biomaterials science and engineering*. (Springer Science & Business Media, 2012).

[2] Manivasagam, G., Dhinasekaran, D. & Rajamanickam, A. Biomedical Implants: Corrosion and its Prevention-A Review. *Recent Patents on Corrosion Science* (2010).

[3] Levy, S. B. & Marshall, B. Antibacterial resistance worldwide: causes, challenges and responses. *Nat Med* (2004). 10.1038/nm1145 15577930

[4] Campoccia D, Montanaro L, Arciola CR (2013). A review of the biomaterials technologies for infection-resistant surfaces. Biomaterials.

[5] Magill SS (2014). Multistate Point-Prevalence Survey of Health Care–Associated Infections. New England Journal of Medicine.

[6] Ceroni D (2016). From prevention of pin-tract infection to treatment of osteomyelitis during paediatric external fixation. Journal of Children’s Orthopaedics.

[7] Kazmers NH, Fragomen AT, Rozbruch SR (2016). Prevention of pin site infection in external fixation: a review of the literature. Strategies in Trauma and Limb Reconstruction.

[8] Shahbaz, M., Chotai, P., Anderson, C. & Clarkson, J. The Effect of Silver on the Reduction of Pin-Tract Infections in Patients Undergoing Hand Surgery: A Retrospective Comparison. *The Internet Journal of Hand Surgery* **6** (2014).

[9] Song Z (2013). Prosthesis Infections after Orthopedic Joint Replacement: The Possible Role of BacterialBiofilms. Orthopedic Reviews.

[10] Veerachamy S, Yarlagadda T, Manivasagam G, Yarlagadda PK (2014). Bacterial adherence and biofilm formation on medical implants: a review. Proceedings of the Institution of Mechanical Engineers, Part H: Journal of Engineering in Medicine.

[11] Tlaskalová-Hogenová H (2004). Commensal bacteria (normal microflora), mucosal immunity and chronic inflammatory and autoimmune diseases. Immunology Letters.

[12] de Breij A (2016). Prevention of Staphylococcus aureus biomaterial-associated infections using a polymer-lipid coating containing the antimicrobial peptide OP-145. Journal of Controlled Release.

[13] Harris LG, Richards RG (2006). Staphylococci and implant surfaces: a review. Injury.

[14] Kertzman Z (2008). Mechanical, tribological, and biocompatibility properties of ZrN‐Ag nanocomposite films. Journal of Biomedical Materials Research Part A.

[15] Wickens DJ (2012). Antimicrobial activity of nanocomposite zirconium nitride/silver coatings to combat external bone fixation pin infections. International Journal of Artificial Organs.

[16] Kelly P (2010). Comparison of the tribological and antimicrobial properties of CrN/Ag, ZrN/Ag, TiN/Ag, and TiN/Cu nanocomposite coatings. Surface and Coatings Technology.

[17] Wickens D (2014). Quantifying the pattern of microbial cell dispersion, density and clustering on surfaces of differing chemistries and topographies using multifractal analysis. Journal of Microbiological Methods.

[18] Xiu Z-M, Ma J, Alvarez PJ (2011). Differential effect of common ligands and molecular oxygen on antimicrobial activity of silver nanoparticles versus silver ions. Environmental Science & Technology.

[19] Mijnendonckx K, Leys N, Mahillon J, Silver S, Van Houdt R (2013). Antimicrobial silver: uses, toxicity and potential for resistance. Biometals.

[20] Rai M, Yadav A, Gade A (2009). Silver nanoparticles as a new generation of antimicrobials. Biotechnology Advances.

[21] Jung WK (2008). Antibacterial activity and mechanism of action of the silver ion in Staphylococcus aureus and Escherichia coli. Applied and Environmental Microbiology.

[22] Atiyeh BS, Costagliola M, Hayek SN, Dibo SA (2007). Effect of silver on burn wound infection control and healing: review of the literature. Burns.

[23] Hobman JL, Crossman LC (2015). Bacterial antimicrobial metal ion resistance. Journal of medical microbiology.

[24] Liau SY, Read DC, Pugh WJ, Furr JR, Russell AD (1997). Interaction of silver nitrate with readily identifiable groups: relationship to the antibacterial action of silver ions. Letters in Applied Microbiology.

[25] Feng Q (2000). A mechanistic study of the antibacterial effect of silver ions on Escherichia coli and Staphylococcus aureus. Journal of Biomedical Materials Research.

[26] Castellano JJ (2007). Comparative evaluation of silver‐containing antimicrobial dressings and drugs. International Wound Journal.

[27] Raimondi F, Scherer GG, Kötz R, Wokaun A (2005). Nanoparticles in energy technology: examples from electrochemistry and catalysis. Angewandte Chemie International Edition.

[28] Song H, Ko K, Oh I, Lee B (2006). Fabrication of silver nanoparticles and their antimicrobial mechanisms. European Cells and Materials Journal.

[29] Kelly P (2009). A study of the antimicrobial and tribological properties of TiN/Ag nanocomposite coatings. Surface and Coatings Technology.

[30] Whitehead KA, Verran J (2006). The effect of surface topography on the retention of microorganisms. Food and Bioproducts Processing.

[31] Alla RK (2011). Surface roughness of implants: a review. Trends in Biomaterials and Artificial Organs.

[32] Rhim JW, Wang LF, Hong SI (2013). Preparation and characterization of agar/silver nanoparticles composite films with antimicrobial activity. Food Hydrocolloids.

[33] Cho K-H, Park J-E, Osaka T, Park S-G (2005). The study of antimicrobial activity and preservative effects of nanosilver ingredient. Electrochimica Acta.

[34] Sandström, S. The antibacterial effect of silver with different release kinetics. (2011).

[35] Vaidya MY, McBain AJ, Butler JA, Banks CE, Whitehead KA (2017). Antimicrobial Efficacy and Synergy of Metal Ions against Enterococcus faecium, Klebsiella pneumoniae and Acinetobacter baumannii in Planktonic and Biofilm Phenotypes. Scientific Reports.

[36] Gough, L. P., Shacklette, H. T. & Case, A. A. Element concentrations toxic to plants, animals, and man. (US Govt. Print. Off., 1979).

[37] Saldaña L (2007). *In vitro* biocompatibility of an ultrafine grained zirconium. Biomaterials.

[38] Möller B (2012). A comparison of biocompatibility and osseointegration of ceramic and titanium implants: an *in vivo* and *in vitro* study. International Journal of Oral and Maxillofacial Surgery.

[39] Sollazzo V (2008). Zirconium oxide coating improves implant osseointegration *in vivo*. Dental Materials.

[40] Akagawa Y, Ichikawa Y, Nikai H, Tsuru H (1993). Interface histology of unloaded and early loaded partially stabilized zirconia endosseous implant in initial bone healing. The Journal of Prosthetic Dentistry.

[41] Pauksch L (2014). Biocompatibility of silver nanoparticles and silver ions in primary human mesenchymal stem cells and osteoblasts. Acta Biomaterialia.

[42] Silver S, Phung LT, Silver G (2006). Silver as biocides in burn and wound dressings and bacterial resistance to silver compounds. Journal of Industrial Microbiology and Biotechnology.

[43] Wilkinson L, White R, Chipman J (2011). Silver and nanoparticles of silver in wound dressings: a review of efficacy and safety. Journal of Wound Care.

[44] Bergin, S. & Wraight, P. Silver based wound dressings and topical agents for treating diabetic foot ulcers. *The Cochrane Library* (2006). 10.1002/14651858.CD005082.pub2 16437516

[45] Ip M, Lui SL, Poon VK, Lung I, Burd A (2006). Antimicrobial activities of silver dressings: an *in vitro* comparison. Journal of Medical Microbiology.

[46] Aziz Z, Abu S, Chong N (2012). A systematic review of silver-containing dressings and topical silver agents (used with dressings) for burn wounds. Burns.

[47] Heyneman A, Hoeksema H, Vandekerckhove D, Pirayesh A, Monstrey S (2016). The role of silver sulphadiazine in the conservative treatment of partial thickness burn wounds: A systematic review. Burns.

[48] Perez E (2014). Microbial biofilms on needleless connectors for central venous catheters: comparison of standard and silver-coated devices collected from patients in an acute care hospital. Journal of Clinical Microbiology.

[49] Boyce JM (2016). Modern technologies for improving cleaning and disinfection of environmental surfaces in hospitals. Antimicrobial Resistance & Infection Control.

[50] Masse A (2000). Prevention of pin track infection in external fixation with silver coated pins: clinical and microbiological results. Journal of Biomedical Materials Research.

[51] Williams S (1998). Distinguishing between living and nonliving bacteria: evaluation of the vital stain propidium iodide and its combined use with molecular probes in aquatic samples. Journal of Microbiological Methods.

[52] Radzig M (2013). Antibacterial effects of silver nanoparticles on gram-negative bacteria: influence on the growth and biofilms formation, mechanisms of action. Colloids and Surfaces B: Biointerfaces.

[53] Marambio-Jones C, Hoek EM (2010). A review of the antibacterial effects of silver nanomaterials and potential implications for human health and the environment. Journal of Nanoparticle Research.

[54] Noyce J, Michels H, Keevil C (2006). Potential use of copper surfaces to reduce survival of epidemic meticillin-resistant Staphylococcus aureus in the healthcare environment. Journal of Hospital Infection.

[55] Cappelier J, Lazaro B, Rossero A, Fernandez-Astorga A, Federighi M (1997). Double staining (CTC-DAPI) for detection and enumeration of viable but non-culturable Campylobacter jejuni cells. Veterinary Research.

[56] Bhupathiraju VK, Hernandez M, Landfear D, Alvarez-Cohen L (1999). Application of a tetrazolium dye as an indicator of viability in anaerobic bacteria. Journal of Microbiological Methods.

[57] Da Silva EP, De Martinis ECP (2013). Current knowledge and perspectives on biofilm formation: the case of Listeria monocytogenes. Applied Microbiology and Biotechnology.

[58] Laflamme C, Lavigne S, Ho J, Duchaine C (2004). Assessment of bacterial endospore viability with fluorescent dyes. Journal of Applied Microbiology.

[59] Oss, C. v., Good, R. & Busscher, R. Estimation of the polar surface tension parameters of glycerol and formamide, for use in contact angle measurements on polar solids. *Journal of Dispersion Science and Technology* **11**, 75–81 (1990).

[60] van Oss CJ (1995). Hydrophobicity of biosurfaces — Origin, quantitative determination and interaction energies. Colloids and Surfaces B: Biointerfaces.

[61] Van Oss C, Good R, Chaudhury M (1986). The role of van der Waals forces and hydrogen bonds in “hydrophobic interactions” between biopolymers and low energy surfaces. Journal of Colloid and Interface Science.

