# Author Correction: Antimicrobial activity of Ti-ZrN/Ag coatings for use in biomaterial applications

**DOI:** 10.1038/s41598-018-24174-9

**Published:** 2018-04-16

**Authors:** Anthony J. Slate, David J. Wickens, Mohamed El Mohtadi, Nina Dempsey-Hibbert, Glen West, Craig E. Banks, Kathryn A. Whitehead

**Affiliations:** 1 Microbiology at Interfaces Group, School of Healthcare Sciences, Manchester Metropolitan University, Chester Street, Manchester, M1 5GD UK; 2 School of Science and the Environment, Manchester Metropolitan University, Chester Street, Manchester, M1 5GD UK; 3 ESR Technology, Birchwood Park, Warrington, UK; 4 Surface Engineering Group, School of Engineering, Manchester Metropolitan University, Manchester, M1 5GD UK

Correction to: *Scientific Reports* 10.1038/s41598-018-20013-z, published online 24 January 2018

Glen West was omitted from the author list in the original version of this Article. This has been corrected in the PDF and HTML versions of the Article.

The Acknowledgements section now reads:

“This work was funded by Manchester Metropolitan University.”

The Author Contributions section now reads:

“A.J. Slate wrote the manuscript. D.J. Wickens and M. El Mohtadi were involved in the experimental protocols. C.E. Banks and N. Dempsey-Hibbert were involved in the formulation of the manuscript and undertook supervisory roles. G. West oversaw the coating production. K.A. Whitehead also wrote the manuscript and conceived the concept and ideas presented in this paper.”

In addition, the original version of this Article omitted an affiliation for G. West. The correct affiliations are listed below and have been corrected in the PDF and HTML versions of the Article:

Microbiology at Interfaces Group, School of Healthcare Sciences, Manchester Metropolitan University, Chester Street, Manchester, M1 5GD, UK

School of Science and the Environment, Manchester Metropolitan University, Chester Street, Manchester, M1 5GD, UK

ESR Technology, Birchwood Park, Warrington, UK

Surface Engineering Group, School of Engineering, Manchester Metropolitan University, Manchester, M1 5GD, UK

